# Calibration of Voltage Transformers and High- Voltage Capacitors at NIST

**DOI:** 10.6028/jres.094.019

**Published:** 1989

**Authors:** William E. Anderson

**Affiliations:** National Institute of Standards and Technology, Gaithersburg, MD 20899

**Keywords:** calibration, capacitors, dissipation factor, electric power, electrical standards, NIST services, voltage transformers

## Abstract

The National Institute of Standards and Technology (NIST) calibration service for voltage transformers and high-voltage capacitors is described. The service for voltage transformers provides measurements of ratio correction factors and phase angles at primary voltages up to 170 kV and secondary voltages as low as 10 V at 60 Hz. Calibrations at frequencies from 50–400 Hz are available over a more limited voltage range. The service for high-voltage capacitors provides measurements of capacitance and dissipation factor at applied voltages ranging from 100 V to 170 kV at 60 Hz depending on the nominal capacitance. Calibrations over a reduced voltage range at other frequencies are also available. As in the case with voltage transformers, these voltage constraints are determined by the facilities at NIST.

## 1. Introduction

This paper describes the National Institute of Standards and Technology (NIST) methodology for calibrating high-voltage capacitors and transformers. This should benefit NIST clients in several ways. First, by understanding how NIST makes these measurements, the clients might be able to define weaknesses in their own measurement procedures and correct them. Second, the clients should be able to make better use of the data in the calibration report (e.g., to understand what is meant by the uncertainty statement). Third, the clients should be able to better specify the required test conditions so that information more pertinent to their needs can be obtained at a lower cost.

This paper describes two different calibration services: high-voltage capacitors and voltage transformers. At NIST these two services are performed using the same equipment. In fact, in order to calibrate a voltage transformer, one of the steps is to measure the ratio of two capacitors. The two services are therefore discussed in parallel.

There are several different ways to measure the ratio and phase angle of a voltage transformer. Harris [1] categorizes them as the direct versus comparative methods and within these two classifications either the deflection or null measurement technique. A direct measurement is defined here as a measurement in which the quantity of interest can be determined without a comparison to some absolute standard.

In the “direct deflection method” the primary and secondary voltage vectors are each directly measured. This approach is, in general, of most value for lower voltage transformers (i.e., primary voltages of order 100 V). Even then more accurate, less difficult measurements can be made using one of the other techniques.

In the past NIST had used a “comparative null method” to calibrate voltage transformers. The unknown transformer was compared to a NIST reference transformer using a voltage comparator consisting of a variable resistive divider and a mutual inductor. Reference transformers were available with ratios ranging from 1/1 up to 2000/1. Measurement uncertainties in the comparison of the unknown transformer with the reference transformer were ±0.01% for ratio and ±0.3 minutes for phase angle. The ratio and phase angle of the reference transformers were known to about the same accuracy. There are several disadvantages to this approach. Since the comparator has a limited range, several reference transformers must be available to cover the anticipated users’ needs. The ratio and phase angles of each one of these transformers must be carefully determined over the secondary voltage range of interest. These transformers then have to be rechecked at regular intervals to determine if the ratios and phase angles have changed.

If a direct measurement method were available that was sufficiently accurate and straightforward to make the calibration of these reference transformers a simple task, then that method could be used to measure the client’s transformer directly. At NIST, the “direct null method” in use originally involved balancing the secondary of the reference transformer against the output of a resistive divider used in conjunction with a variable mutual inductor to provide phase angle balance. Such a measurement was difficult because the resistive divider ratio changed with heating. Since the late 1960s a “direct null method” has been available that is straightforward and accurate and is now used at NIST in place of comparative methods using reference transformers.

**Figure 1:**
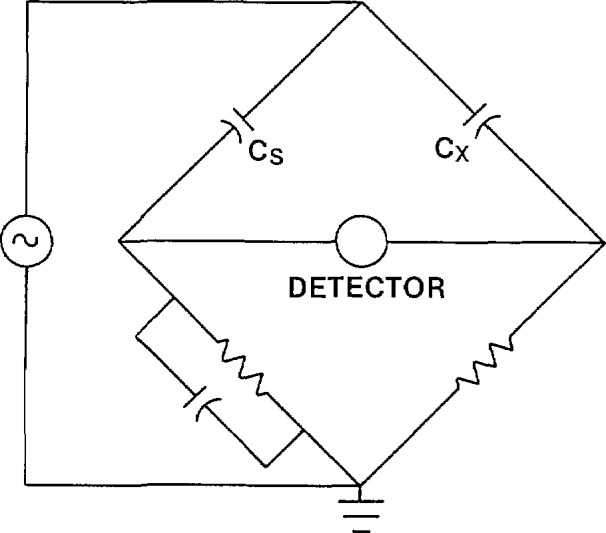
Schering bridge.

Capacitors are invariably measured by balancing the unknown capacitor against a known standard using some type of bridge arrangement. There are a variety of such bridges described in the literature [2]. The one most used in high-voltage applications in the last 60 years is the Schering bridge (fig. 1). The two high-voltage arms of this bridge consist of the standard and unknown capacitors. The two low voltage arms are resistors (one has a parallel capacitor for phase angle balance).

The main limitation of the Schering bridge is that the low side of the unknown and standard capacitors are not at ground potential at bridge balance. Therefore, without carefully guarding the bridge components, stray currents can affect the bridge accuracy. The voltage applied to the shields to eliminate these stray currents must be adjusted for both magnitude and phase. Unfortunately this procedure is not perfect and bridge accuracy is consequently affected. Another limitation of the Schering bridge is the inherent inaccuracy of the resistance ratio of the two low-voltage arms.

The current comparator bridge developed by Kusters and Petersons [3] allows the intercomparison of two capacitors with their low-voltage terminals at ground potential, thereby eliminating the main objection in using the Schering bridge. This bridge, used in both voltage transformer and capacitor calibrations, will be described in some detail in section 4. There is an important distinction between the calibration of voltage transformers and capacitors at NIST. The voltage transformer calibration is of the direct null type, and the capacitor calibration is of the comparative null type. In other words, the accuracy of the capacitance measurements ultimately depends on the uncertainty in assigning a value to a standard capacitor. The standard capacitor used in this service is directly traceable to the calculable cross capacitor [4] which, in turn, is known in terms of the fundamental unit of length.

The remainder of this paper is divided into the following subject areas: voltage transformers and capacitors covered by the service, measurement methodology, measurement instrumentation, and analysis of uncertainties. The contents of this paper plus the cited references should provide the reader with a fairly complete description of the voltage transformer and high-voltage capacitor calibration services at NIST.

## 2. Range of Services

The NIST measurement capabilities are summarized in table 1 and discussed in more detail below.

### 2.1 Voltage Transformers

Presently, voltage transformers (assuming they are of sufficient quality to be used as laboratory standards) with primary voltages up to 170 kV at a frequency of 60 Hz can be calibrated at NIST. This maximum voltage is imposed by the supply transformer and not by limitations in the measurement instrumentation. Therefore, this constraint should not be considered rigid and clients should contact the NIST about present physical limitations.

The largest portion of the voltage transformers submitted to NIST are calibrated with total estimated uncertainties of ±300 parts per million (ppm) in ratio, and ±0.3 mrad in phase angle. These transformers are of sufficient quality to be considered transfer standards. Historically these transformers have shown excellent long-term stability, rarely changing by more than 100 ppm in ratio or 0.1 mrad in phase (at or below rated burden) for periods as long as 30 years or more. In general, the voltage and burden dependence of these transformers are the major contributors to the measurement uncertainties. These uncertainties (±300 ppm for ratio, ±0.3 mrad for phase angle) meet the accuracy requirements of most NIST clients.

Voltage transformers of a higher accuracy class often serve as transfer standards for manufacturers of voltage transformers and voltage transformer test sets (voltage comparators). The estimated uncertainties for these transformers are ±100 ppm in ratio, and *±*0.1 mrad in phase angle. They are generally designed for use with very small burdens (<15 volt-amperes).

The above discussion for voltage transformers assumes a voltage at a frequency of 60 Hz. The National Institute of Standards and Technology has some capability to calibrate voltage transformers from about 50 Hz to 400 Hz (at the lower voltage and power ranges). Such calibrations are infrequent and clients interested in these voltage ranges and measurement uncertainties should contact NIST directly.

### 2.2 Capacitors

The maximum voltage for capacitor calibrations is presently 170 kV at 60 Hz. The restrictions are imposed by the supply transformer and not by limitations in the measurement instrumentation. Therefore, this constraint should not be considered time invariant and clients should contact NIST about present physical limitations.

The maximum power available is 50 kVA (i.e., *C*<50,000/{27π60*V*^2^} where *V* is the applied voltage and *C* is the capacitance). In order to energize the capacitors a resonant circuit is often required to couple the necessary energy into the client’s capacitor. Since this requires the availability of an assortment of series and parallel inductors and capacitors, there are undoubtedly some capacitors that, despite having a burden of less than 50 kVA, cannot be calibrated. The client should contact NIST before submitting a capacitor for calibration. As with voltage transformers, NIST restricts its calibration services to those devices of sufficient quality to be used as transfer standards. This in general depends upon the stability of the capacitor (i.e., whether the measured capacitance and dissipation factor are intrinsic properties of the device itself or instead are largely a function of conditions at the time of the calibration). For example, small two-terminal capacitors (less than 10,000 pF) may be significantly influenced by stray capacitance in the measurement circuit. There are cases, however, where one component (capacitance or dissipation factor) is stable and the other is not. For example, power factor capacitors often have relatively stable dissipation factors but have capacitances that vary significantly with applied voltage (even demonstrating hysteresis effects) and temperature. In this case a calibration of dissipation factor would be meaningful. It also is important that the capacitors have connectors^1^ that are generally available, e.g., BNC, GR, UHF, BPO, or Type N.

The most accurate capacitor calibrations have an uncertainty of ±25 ppm for capacitance and an uncertainty of ±5×10^−6^ for dissipation factor. For capacitors with large dissipation factors, the dissipation factor uncertainty is generally at least ±1% of the measured value ±5×10^−6^. The uncertainty in the capacitance value and the dissipation factor can be largely a function of the stability of the capacitor.

## 3. Measurement Methodology

### 3.1 Basic Measurement Circuits

**Figure 2:**
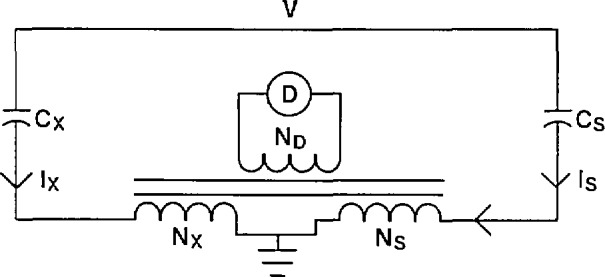
Basic measurement circuit for the calibration of a high-voltage capacitor.

The current comparator bridge used to calibrate voltage transformers and high-voltage capacitors will be discussed in considerable detail in section 4. A brief discussion of this bridge will be presented here in order to facilitate understanding of the NIST measurement methodology. A simplified circuit for measuring the ratio of two capacitors is shown in figure 2. (The active circuitry to achieve dissipation factor balance is not included.) At balance

$$\frac{V2\pi fC_{x}N_{x}}{N_{D}}=\frac{V2\pi fC_{s}N_{s}}{N_{D}},$$ (1)

where *f* is the frequency. This can be rewritten

$$C_{x}=\frac{N_{s}}{N_{x}}C_{s}.$$ (2)

**Figure 3:**
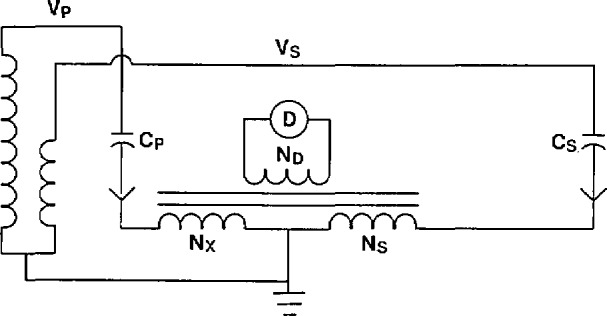
Basic measurement circuit for the calibration of a voltage transformer.

The simplified circuit for measuring the ratio of voltage transformers is shown in figure 3. At balance

$$\frac{V_{p}2\pi fC_{p}N_{x}}{N_{d}}=\frac{V_{s}2\pi fC_{s}N_{s}}{N_{d}}$$ (3)

or,

$$\frac{V_{p}}{V_{s}}=\frac{N_{s}C_{s}}{N_{x}C_{p}}.$$ (4)

The ratio of the two capacitors in eq (2) can be measured using the circuit of figure 2.

The measurement of a voltage transformer or a capacitor both involve the measurement of the ratio of two standard capacitors. The measurement of capacitors will be discussed below followed by a discussion on the measurement of voltage transformers.

### 3.2 Capacitors

#### 3.2.1 General Measurement Technique

**Figure 4:**
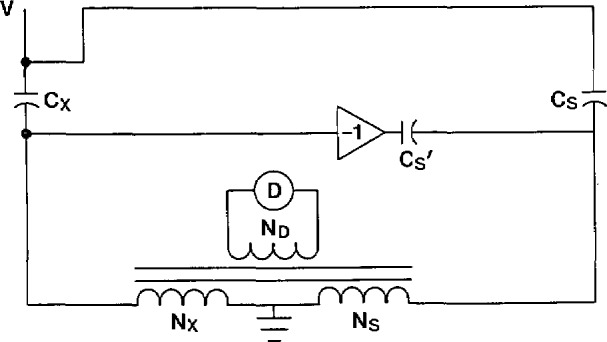
Basic measurement circuit for the four-terminal calibration of large capacitors.

Capacitors are measured by balancing the current through the capacitor under test against the current through a standard air or compressed gas capacitor as shown in figure 2. Large capacitors (> 1 *μ*F) necessitate a four-terminal measurement as shown in figure 4. This measurement will be discussed in section 4. The four-terminal measurement eliminates the effect of leads in the measurement of capacitance and dissipation factor.

#### 3.2.2 Information Necessary to Initiate Calibration

The client usually only needs to specify the voltage and the frequency. For small capacitors (10,000 pF or less), it is essential that the low-voltage electrode and the conductor leading to the measurement instrumentation be shielded by a grounded conductor. Otherwise, the stray capacitance may cause significant measurement error. The National Institute of Standards and Technology requires some sort of standard connector (BNC, UHF, GR, BFO, or Type N) at the low-voltage terminal in order to connect to the measurement system. Larger capacitors do not need to be shielded but must be measured as a four terminal admittance because of the non-negligible lead impedance. A description of how this measurement is done will be covered in section 4. Capacitors must be stable and reproducible in order to be considered standards and hence warrant a NIST calibration. Power factor capacitors (large capacitors used to tune distribution lines, etc.) are often special cases. Their dissipation factors (in-phase component of the current divided by quadrature component) are often quite stable but their capacitance values are often not. Because of the importance of these capacitors to the electrical industry, they are often acceptable for calibration even though they do not meet normal stability requirements.

Although the instrumentation has been used to calibrate a million-volt standard capacitor at rated voltage, the instrumentation does impose some limitations on the voltage applied to the capacitor. The only limitation on the maximum voltage is that the current through the standard capacitor should be no larger than 10 mA. In order to have reasonable sensitivity, the current should be at least 10 *μ*A. The current through the client’s capacitor can range from 10 *μ*A to 1000 A.

#### 3.2.3 Voltage Dependence

For the calibration of both capacitors and voltage transformers, the voltage coefficient of the standard capacitor is important. The unit of capacitance at NIST is maintained at low voltage. This value must be transferred to the high-voltage standard capacitors at their working voltages. At NIST, considerable work was done to modify a commercial high-voltage standard capacitor to minimize its voltage coefficient and to determine the magnitude of that voltage coefficient [5]. The National Institute of Standards and Technlogy was able to demonstrate that, if care is taken, a well-designed standard capacitor should change capacitance by only a few ppm from 0 to 300 kV. A more recent paper also discusses the problem of the voltage dependence of standard capacitors and describes an international comparison of high-voltage capacitor measurements [6]. (This paper also discusses the effect of shipping and handling on the measured capacitance of a commercial standard capacitor.) The voltage dependence of a compressed gas capacitor principally arises from the coulombic attraction of the two electrodes and is hence quadratic in nature. The capacitor should be expected to vary only slightly at lower voltages. Therefore, a capacitor rated at 200 kV should be quite effective in measuring the voltage dependence of another capacitor rated at 20 kV.

#### 3.2.4 Temperature Dependence

Another concern is the temperature dependence of the high-voltage standard capacitor. The typical dependence is about +20 ppm/°C. This dependence arises solely from the thermal expansion of the components of the capacitor. Since *C* is directly proportional to the electrode area and inversely proportional to the electrode separation, the thermal coefficient of the standard capacitor is proportional to the linear coefficient of expansion. Although the laboratories at NIST are fairly stable in temperature, the comparison of the high-voltage standard capacitor to the low-voltage standard (which has a thermal coefficient of 2 ppm/°C) is done at the beginning and conclusion of the measurement process. The average value is then used in order to minimize the problem associated with this thermal drift.

#### 3.2.5 Gas-Density Dependence

Compressed gas standard capacitors can have an additional source of error associated with gas leakage. Values of *∂C/∂P* (to first order in pressure) measured at a temperature of 22.8 °C are shown in table 2 for three different gases [6].

The gas pressure, *P*, is in units of pascals and the capacitance in picofarads. For a 100-pF capacitor with SF_6_ as the dielectric gas, a 1-psi (6900-Pa) leak would cause the capacitance to decrease by about 140 ppm. It must be stressed that this change is valid only if the pressure change is caused by the loss of gas and not by the lowering of the gas temperature. As can be seen in table 2, the gas density coefficient is largest for SF_6_. Clients using compressed gas capacitors for standards might be advised to monitor the gas pressure with a good quality pressure gauge. Leaking SF_6_-filled capacitors should be checked often against a good low-voltage standard.

### 3.3 Voltage Transformers

#### 3.3.1 Information Necessary to Initiate Calibration

In order to calibrate a voltage transformer, several different parameters must be specified: frequency; windings and/or range; secondary voltage; and burden or impedance across the secondary winding. In some cases, for example when there is a tertiary winding, additional parameters may be required.

#### 3.3.2 Labeling of Terminals

There are some standard conventions as to which of the primary and secondary taps are to be at low or ground potential and which are to be at rated voltage. Some transformers have one tap of the secondary and one tap of the primary winding marked by a “±”. These two taps are connected together and to ground potential. Some transformers use the designators H1, H2 for the primary taps, and X1, X2 (and Y1 and Y2 for the transformers with two secondaries) for the secondary taps. Sometimes the secondary winding has a third tap, X3. By convention the primary and secondary taps with the largest number are connected together and to ground. If the client wants some other arrangement, NIST should be notified prior to the calibration.

#### 3.3.3 Load Imposed by NIST Measurement System

**Figure 5:**
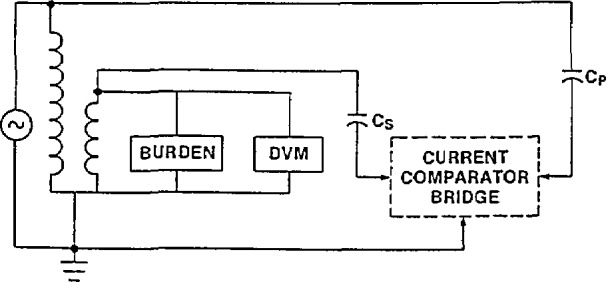
Basic measurement circuit for the calibration of a voltage transformer with a digital voltmeter (DVM) and secondary burden.

The basic measurement circuit is shown in figure 5. The two capacitors shown are three-terminal standard capacitors. Their dissipation factors are typically less than 5 × 10^−6^. The capacitor connected to the secondary usually has the nominal value of 1000 pF. Therefore, for 60-Hz measurements, the capacitor imposes a negligible load (2.7 MΩ or 0.005 volt-amperes at 120 V) on the voltage transformer. Negligible in this case means that the effect of this burden on the measured ratio and phase angle can not be observed at the ppm level. The digital voltmeter (DVM) in figure 5 has an estimated uncertainty of less than ±0.5% of the reading and measures true-rms ac volts. The internal impedance of the DVM is equal to or greater than one megohm.

#### 3.3.4 Possible Errors Caused by Improper Wiring

**Figure 6:**
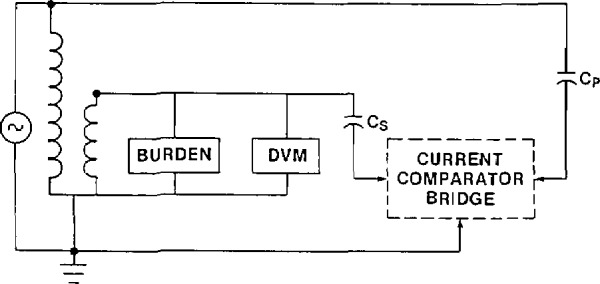
Measurement circuit for the calibration of a voltage transformer. Connection of low-voltage capacitor as shown is incorrect.

The wiring of the circuit shown in figure 5 is critical. For example, it is important that the two capacitors be connected directly to the primary and secondary terminals of the transformer. Consider instead figure 6. The capacitor *C*_s_ is connected to the burden and the DVM instead of directly to the secondary terminal of the transformer. If the secondary burden were an ANSI standard burden ZZ (36 Ω at 120 V, see table 3) and the resistance of the lead connecting the burden to the transformer were 10 mil, the incorrect wiring shown in figure 6 would cause a error in the transformer ratio measurement of about 0.03%. For higher impedance burdens this becomes less of a problem but, in general, one must take precautions to avoid including the voltage drop in the lead connecting the transformer to the burden as part of the voltage on the transformer secondary winding to be measured.

**Figure 7:**
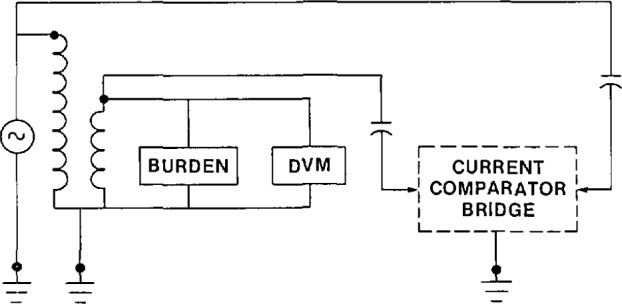
Measurement circuit for the calibration of a voltage transformer. Grounds are poorly defined.

Another major concern in the measurement of the ratio and phase angle of a voltage transformer is the proper definition of the ground point and the avoidance of ground loops. This can best be illustrated by a few examples. In figure 7, some common mistakes are shown. The transformer is energized in such a manner that significant current is forced to flow between the transformer ground and the circuit ground. The resulting voltage drop in the lead connecting the transformer and ground will be part of the ratio and phase angle measured. The high-voltage capacitor is not connected directly to the primary of the transformer under test. The measurement of the ratio and phase angle, therefore, includes the effect of the voltage drop in the lead between the point where the capacitor is connected to the power source and the transformer. In addition, as there are three different “ground” points in the circuit and it is not, in general, possible to know the voltages and impedances between these points, a measurement error is probable.

**Figure 8:**
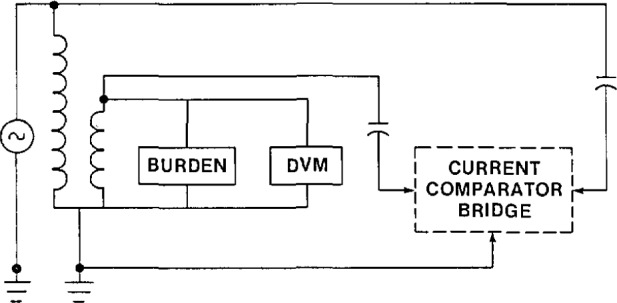
Measurement circuit for the calibration of a voltage transformer. Measurement ground is defined. Transformer excitation current flows from the measurement ground to building ground.

In figure 8 the problem has been eliminated by defining the low-voltage terminal of the transformer as ground. Although this point may significantly differ from the building or utility ground, from the measurement point of view this is the correct ground. It is important that the shields of the three-terminal capacitors, the bridge detector ground, and all other measurement grounds each be connected directly to this point.

In figure 5 the preferred method of wiring a voltage transformer calibration circuit is shown. The client’s transformer is connected in such a way that the energizing current does not flow between the transformer and the measurement ground. All measurement grounds are connected to the transformer ground point. The two capacitors are connected directly to the primary and secondary terminals of the transformer. Only one ground is used in the circuit. While it is not always possible to connect the transformer as in figure 5, this is the best choice. Otherwise tests are required to ensure that systematic errors are not compromising the measurement results.

#### 3.3.5 Burdens

The burden attached to the secondary of the client’s transformer (as shown in fig. 5) is specified by the client. In general this would not be the burden corresponding to the maximum volt-ampere rating of the transformer but instead would be equal to the burden attached to the transformer in its intended use. For example, if the transformer will only have a digital voltmeter attached to its secondary, a calibration with a secondary impedance of one megohm would be more useful than one with an ANSI ZZ burden attached. Since the ANSI burdens are often requested, they are summarized in table 3 [7]. By convention these burdens are defined for a frequency of 60 Hz only.

#### 3.3.6 Substitute Burdens

If the client of the service does not send the secondary burden with the transformer, the National Institute of Standards and Technology will provide the burden. It is not practical to have available and adequately characterized all of the anticipated burdens. Fortunately this is not necessary. If the ratio and phase angle of a transformer is known for two different burden values, the ratio and phase angle at any other burden can be calculated (with certain limitations) [8]. A derivation of the formulas relating the ratios and phase angles at zero and some other known burden value are given in the appendix and presented in abbreviated form below.

**Figure 9:**
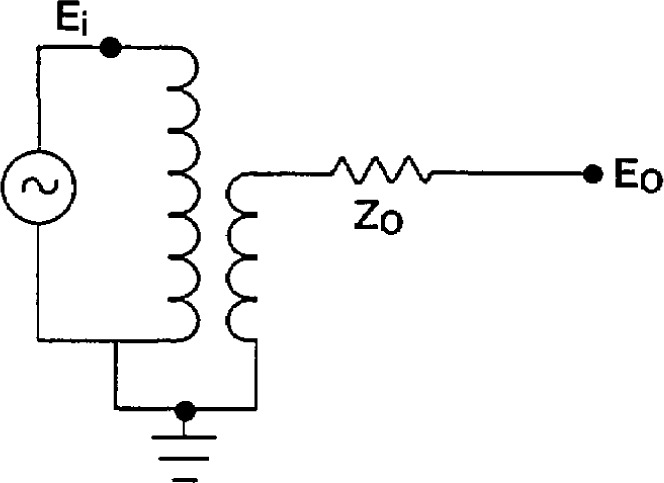
Equivalent circuit of a voltage transformer.

**Figure 10:**
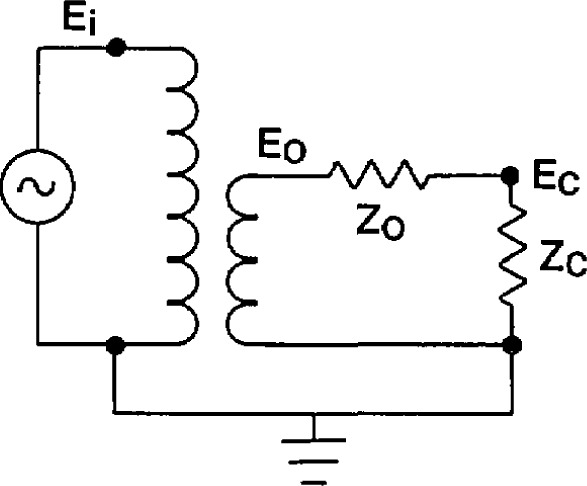
Equivalent circuit of a voltage transformer with secondary burden *Z*_c_.

The voltage transformer will be represented as an ideal transformer with some unknown series output impedance Z_0_, as shown in figure 9. The model has been shown to be sufficiently accurate experimentally. The relationship between the input voltage *E*_i_, and the output voltage with zero burden *E*_0_, is:

$$\frac{E_{i}}{E_{0}}=N\;RCF_{0}e^{-j\Gamma_{0}}=\left|\frac{E_{i}}{E_{0}}\right|\;e^{-j\Gamma_{0}},$$ (5)

where *N* is the nominal (or turns) ratio of the transformer, *RCF* is the ratio-correction factor (*N* × *RCF* = actual ratio) at zero burden, Γ_0_ is the angle by which the secondary voltage vector leads the primary voltage vector and $j=\sqrt{-1}$. A similar relationship exists between the input voltage *E*_i_, and the output voltage *E*_c_, with secondary burden *C* (having impedance *Z*_c_) shown in figure 10:

$$\frac{E_{i}}{E_{c}}=NRCF_{c}e^{-j\Gamma_{c}},$$ (6)

where *RCF*_c_ is the ratio correction factor with secondary burden *C* and Γ_c_ is the corresponding phase angle. If the transformer is measured at zero burden (*RCF*_0_ and Γ_0_) and at burden *T*(*RCF*_t_ and Γ_t_), the ratio correction factor and phase angle at burden C are approximately given by:

$$\begin{array}{l} RCF_{c}\approx RCF_{0}+\frac{B_{c}}{B_{t}}[\left(RCF_{t}-RCF_{0}\right)\\ \;\cos\left(\theta_{t}-\theta_{c}\right)+\left(\Gamma_{t}-\Gamma_{0}\right)\\ \;\sin\left(\theta_{t}-\theta_{0}\right)], \end{array}$$ (7)

where *B*_c_ = 1/*Z*_c_ is the burden in Ω^−1^ of the impedance *Z*_c_, and

$$\begin{array}{l} \Gamma_{c}\approx \Gamma_{0}+\frac{B_{c}}{B_{t}}[\left(\Gamma_{t}-\Gamma_{0}\right)\cos\left(\theta_{t}-\theta_{c}\right)-\left(RCF_{t}-RCF_{0}\right)\\ \;\sin\left(\theta_{t}-\theta_{c}\right)]. \end{array}$$ (8)

The power factor of burden *C* is cos*θ*_c_, *RCF*_c_ is the ratio correction factor calculated for burden *C*, and Γ_c_ is the angle by which the secondary voltage leads the primary voltage for burden *C*.

**Figure 11:**
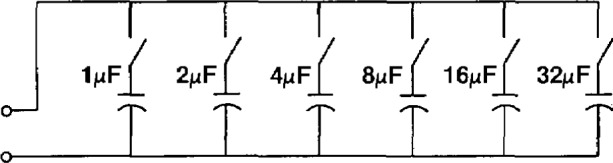
Capacitive burden box.

Equations (7) and (8) can be used to calculate the *RCF* and phase angle for some secondary burden, *C*, if the ratio correction factors and phase angles are known at some other burden *T*, and at zero burden. In practice, at NIST, capacitive burdens are used for the “*T*” or known burdens in eqs (7) and (8). The main reason is their stability. The heat generated in a large resistive burden, for example, is likely to cause the burden’s impedance value to vary. Capacitors, in addition, are compact so even the ZZ burden in table 3 is easy to handle. At NIST, capacitive burden boxes have been constructed in a binary layout (fig. 11) so that capacitors from 1 to 32 *μ*F can be switched in and out allowing any capacitance value from zero to 63 *μ*F. Since a ZZ burden is equivalent to a 74 *μ*F capacitor at 120 V, two such burden boxes are sufficient for nearly all the calibrations at NIST.

Several approximations were made to derive eqs (7) and (8). The approximations relate to the relative ratio of the transformer’s output impedance *Z*_0_ to the impedance of the secondary burden *Z*_t_, or *Z*_c_. The smaller this ratio, the more accurate are eqs (7) and (8). This ratio also affects the differences, *RCF*_t_ *− RCF*_0_ and Γ_t_− Γ_0_. If the ratio correction factor difference is 0.001 or less, and if the phase angle difference is 1 mrad or less than eqs (7) and (8) should be accurate to within ±10 ppm for the ratio correction factor and to within ±10 *μ*rad for the phase angle if it is assumed that the ratio of the burdens is known with no more than ±1 percent uncertainty. Data over the years has indicated that eqs (7) and (8) are always at least that accurate. In order to identify any problems, an extra measurement is made at a different secondary burden to test the predictive capabilities of eqs (7) and (8) for the transformer under test. If a problem is discovered, the error budget is adjusted accordingly.

The above discussion might enable clients of the voltage transformer calibration service to better design their calibration requests. Using eqs (7) and (8), the client might be able to reduce the number of measurements required. A note of caution is in order. It is likely that using a zero burden result and a 10 volt-ampere burden result to predict the transformer’s behavior at a ZZ burden may lead to large inaccuracies. The reasons are twofold. First, the differences *RCF*_t_ *− RCF*_0_ and Γ_t_ − Γ_0_ are likely to be small for a burden as small as 10 volt-amperes and extrapolations can cause large errors. The second reason can be seen from figure 10. The higher current of the ZZ burden will cause *Z*_0_ to heat up and increase in value, leading to errors if eqs (7) and (8) are used. Somewhat better results are likely if one uses a ZZ burden result to predict a transformer’s behavior at 10 volt-amperes. However, it is best to choose burden *T* to have a volt-ampere rating the same order of magnitude as the burden of interest *C.* Also, the values in eqs (7) and (8) are all to be measured at the same frequency and at the same secondary voltage.

#### 3.3.7 Harmonic Effects

The measurement of the ratio and phase angle of a voltage transformer can be affected by the presence of harmonics in the voltage waveform. If a tuned null detector is not used, the balance of a bridge circuit can be difficult in the presence of harmonics and often a precise balance is not possible resulting in increased measurement uncertainties. Harmonics can also lead to errors in measuring the magnitude of the secondary voltage. For example, if an average reading, rms scaled voltmeter measured a 100-V rms fundamental with an in-phase 3-V rms third harmonic, the meter would read 101 V. Setting the voltage to read 100 V on the meter would result in a 1-V discrepancy between the intended and actual voltage. Many transformers have large enough voltage coefficients for this 1-V error in the voltage setting to have a non-negligible effect on the measured ratio correction factor and phase angle. If instead, a true rms voltmeter were used to measure this signal, the measured voltage would be 100.045 V and the resulting error would be negligible. At NIST three different steps are taken to lessen the effects of harmonics. The first is to try to minimize the harmonic content of the power supply. The supply used for most of the calibrations has a total harmonic distortion of order 0.2% of the fundamental. Second, a tuned detector is used to assure that the balance conditions are for the fundamental component of the voltage waveform. And third, all voltage measurements are made with true-rms voltmeters.

#### 3.3.8 Voltage Dependence of Standard Capacitor

An additional measurement concern is the voltage coefficient of the high-voltage standard capacitor shown in figure 5. Although no absolute measurements are required to calibrate a voltage transformer, the ratio of the two standard capacitors must be known. The problem is that the low-voltage standard capacitor typically has a maximum voltage rating of 500 V, and both the primary of the transformer and the high-voltage standard capacitor might be energized to 100 kV. Since the capacitor ratio measurement must be done at less than 500 V, the voltage dependence of the high-voltage capacitor is important. This problem was discussed in section 3.2.

## 4. Measurement Instrumentation

The calibration of voltage transformers and high-voltage capacitors at NIST requires the combined use of standard capacitors and the current comparator bridge. Standard capacitors have been thoroughly discussed in the literature [5, 6, 9,]. The care that must be taken with their use in these types of measurements has been discussed above. The current comparator bridge will be discussed in this section.

**Figure 12:**
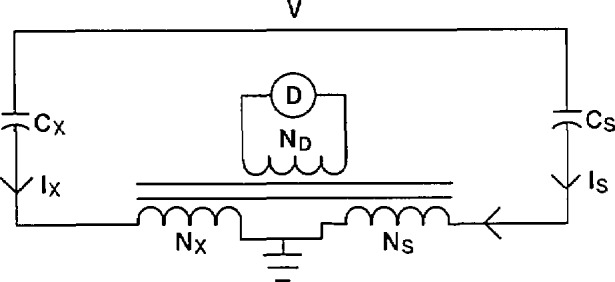
Basic current comparator bridge.

The current comparator bridge can be thought of as a voltage comparator transformer arm bridge in which the detector and power source have been interchanged. Traditionally, the disadvantage of the current comparator bridge versus the voltage comparator bridge is the signal-to-noise level. For high-voltage measurement applications, this is no longer a problem. Kusters and Petersons were the first to develop this bridge for the comparison of two capacitors at high voltage [3]. A basic current comparator bridge is shown in figure 12. The current in the unknown capacitor, *C*_x_, is balanced against the current in the standard capacitor, *C*_s_, by varying the turns ratios, *N*_s_ and *N*_x_.

Balance is achieved when the signal at the detector D is equal to zero. At balance *I*_x_*N*_x_ = *I*_s_*N*_s_ or:

$$V2\pi fC_{x}N_{x}=V2\pi fC_{s}N_{s},$$ (9)

where *f* is the frequency. This balance equation can also be expressed as:

$$C_{x}=\frac{N_{s}}{N_{x}}C_{s}$$ (10).

**Figure 13:**
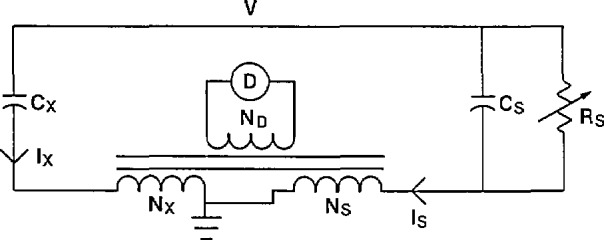
Current comparator bridge with high-voltage resistor for in-phase current balance.

The bridge shown in figure 12 has no means of balancing the in-phase current resulting from a non-ideal unknown capacitor *C*_x_. The current comparator in figure 13 does have the capability of balancing both the in-phase and quadrature components of the capacitive current. The difficulty with the approach used in figure 13 is that the applied high voltage is across the variable resistance *R*_s_. It is nearly impossible to design a stable high-voltage variable resistor with negligible phase angle. Another means is necessary to balance the in-phase current, preferably at low voltage using well-characterized components.

**Figure 14:**
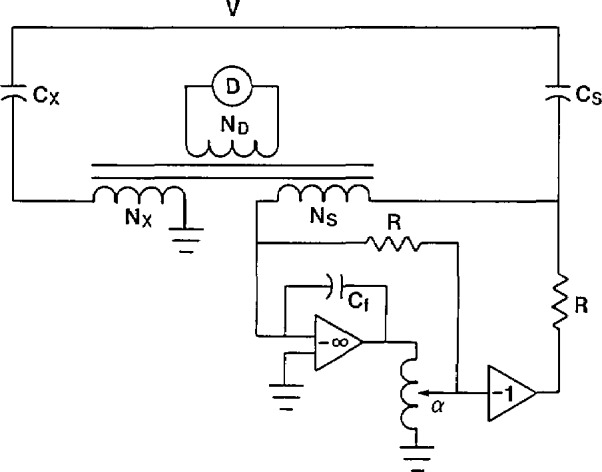
Current comparator with superior in-phase current balance.

The current comparator shown in figure 14 provides a satisfactory means of achieving both the in-phase and quadrature current balances. The quadrature current balance is identical to that in figures 12 and 13 above. The in-phase current balance is accomplished at low voltage with the aid of an operational amplifier. The current from the standard capacitor, after passing through the *N*_s_ winding, goes to the inverting input of the operational amplifier. This point is at virtual ground so the capacitive current balance, eq (10), is not affected. The feedback capacitor *C*_f_ causes the output voltage of the operational amplifier to be a small fraction (*C*_s_/*C*_f_ where *C*_f_ is approximately 10 μF) of the applied voltage and π radians out of phase with it. The inductive voltage divider allows a known fraction, *α*, of this output signal to be applied across a standard resistor *R*. As can be seen from figure 14, the signal is first inverted before the resistor in order to have the correct phase relationship with the unknown in-phase current.

It is necessary that the non-inverted signal be applied to an identical standard resistor as shown in figure 14 so that the current from the standard winding *N*_s_ reaching the operational amplifier has no phase defect. The in-phase current into the standard winding *N_s_* is then equal to:

$$I_{\text{in}}=\frac{\left(\alpha VC_{s}/C_{f}\right)}{R}$$ (11)

Since the quadrature current *I*_out_ = *V2πfC*_s_, the dissipation factor is:

$$\text{DF}=\frac{I_{\text{in}}}{I_{\text{out}}}=\frac{\alpha VC_{s}}{2\pi fRVC_{f}C_{s}}$$ (12)

or

$$\text{DF}=\frac{\alpha }{2\pi fRC_{f}}.$$ (13)

The resistor *R* can be chosen so that *α* is direct reading in percent or milliradians.

**Figure 15:**
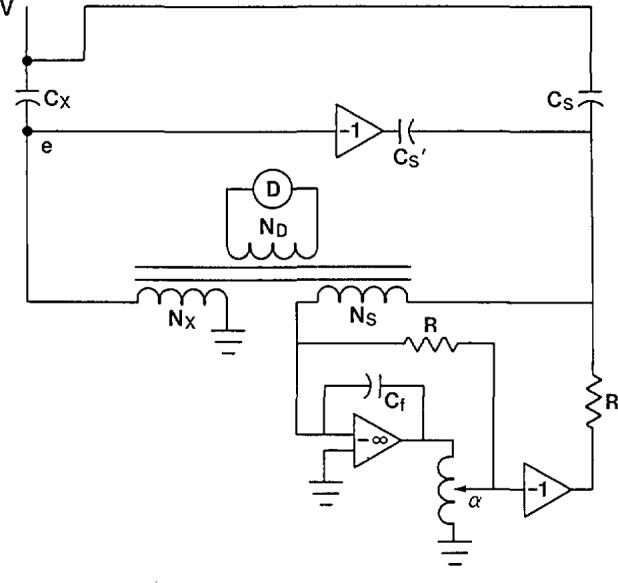
Current comparator bridge modified for four-terminal capacitance measurements.

In some cases, particularly for larger capacitors, it is necessary to make a four-terminal measurement. This is required when the lead and winding impedances become a significant fraction of the impedance to be measured. Figure 15 shows a current comparator bridge with this capability. Because of the non-negligible lead and winding impedance, there is some voltage *e* at the low-voltage terminal of the capacitor. This voltage signal is inverted as shown in figure 15 and connected to the *N*_s_ winding through a capacitor C_s′_. The current through the unknown capacitor is:

$$I_{x}=j2\pi f\left(V-e\right)C_{x}.$$ (14)

The current reaching the *N*_s_ winding is:

$$I_{s}=j2\pi VC_{s}-j2\pi feC_{s^{\prime }}.$$ (15)

If *C*_s′_ is adjusted prior to the measurement to be equal to *C*_s_ then eq (15) reduces to:

$$I_{s}=j2\pi f\left(V-e\right)C_{s}.$$ (16)

Comparing this with eq (14), the effect of the compensation circuit has been to place the same voltage across both the standard and unknown capacitors. This is exactly what is required for lead compensation.

**Figure 16:**
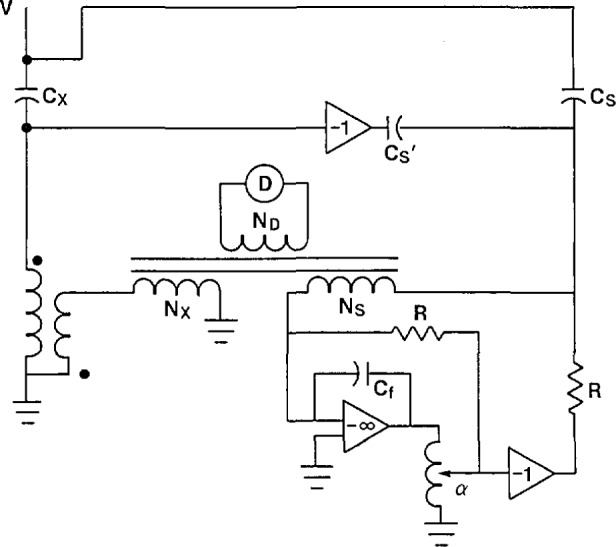
Current comparator bridge with external range extender.

Figure 16 shows the last enhancement of the bridge to be discussed. The National Institute of Standards and Technology’s current comparator bridge has an internal range of 1000:1 (i.e., the maximum value of *N*_d_/*N*_x_ is 1000). The external current transformer shown in figure 16, referred to as a range extender, increases the measurement range by a factor of 1000 allowing the comparison of two currents differing in magnitude by as much as a factor of a million. As with the transformers internal to the current comparator bridge, the accuracy requirements on the range extender are quite stringent. Further details on the design of a ppm current comparator and the specifics of NIST’s current comparator bridge are available in the literature [10, 11].

The current comparator bridge is quite straightforward to use and has proven to be rugged in practice. In order to monitor the behavior of NIST’s current comparator bridge, a check standard is maintained. In this case, the check standard consists of two high quality standard capacitors. The ratio of the two capacitors is measured quarterly. For the last 8 years, this ratio has been stable to within about 20 ppm as can be seen in table 4.

The drift can readily be attributed to the two capacitors. The 9 ppm change between 10/84 and 4/85 occurred apparently after one of the capacitors had been used for another purpose. An independent measurement of that capacitor verified the change. While the use of this check standard cannot prove that the bridge is still working to the ppm level, it can alert the user of changes large enough to affect calibration results. Of course, since the two capacitive currents are largely balanced using stable passive components (i.e., transformer windings), one expects that the bridge should be stable. It should be noted that if a transformer winding were to become open or short circuited the result would be dramatic and readily observed by the operator.

**Figure 17:**
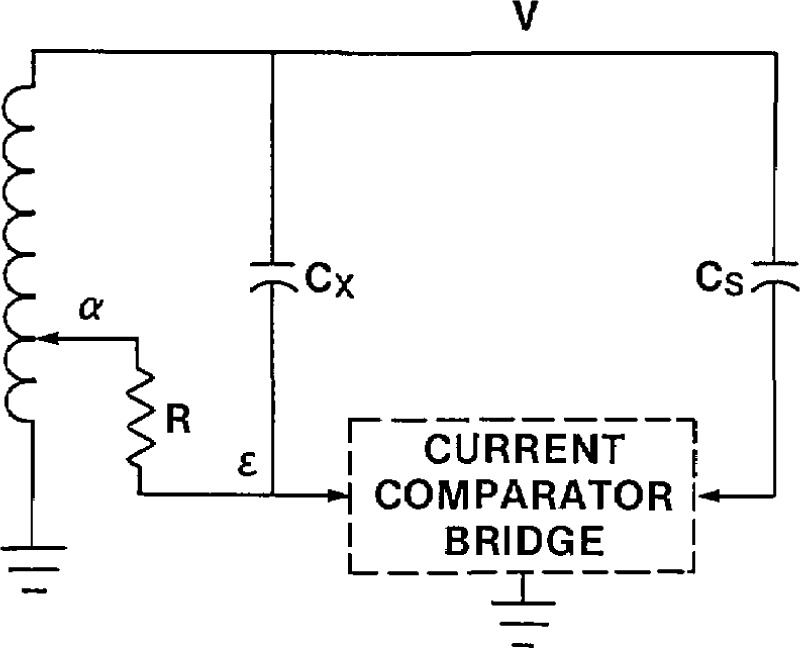
Circuit for checking operation of dissipation factor measurement of current comparator bridge.

The situation with the dissipation factor (or in-phase current) balance is different as active components play an important role. Also, it is difficult to design a stable dissipation factor standard to act as a check standard. This problem has been overcome by using the circuit in figure 17. Standard capacitors are connected to the standard and unknown sides of the bridge. The known in-phase current is applied with the use of the inductive voltage divider and a resistor as shown. The advantage of this circuit is that the voltage across the resistor is small (~0.3 V). However, because of the small voltage, any error voltage, e, at the low side of the resistor, *R*, becomes important. The in-phase current entering the *N*_x_ winding is:

$$I_{\text{in}}=\frac{\alpha V-\epsilon }{R},$$ (17)

where *α* is the ratio of the inductive voltage divider (*α*≪1). The dissipation factor *I*_in_/*I*_out_ is then equal to:

$$DF=\left[\frac{\alpha V-\epsilon }{\left(V-\epsilon \right)R2\pi fC_{x}}\right].$$ (18)

**Figure 18:**
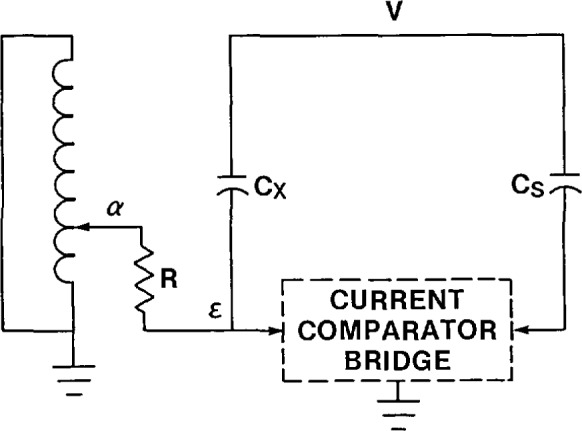
Circuit for checking operation of dissipation factor measurement of current comparator bridge. Input is grounded in order to measure e in eq (18).

The effect of *ϵ* can be significant at the ppm level and needs to be eliminated. The circuit in figure 18 is identical to that in figure 17 except that the input of the inductive voltage divider is grounded. The dissipation factor in this case is then:

$$DF_{0}=\left[\frac{-\epsilon }{\left(V-\epsilon \right)R2\pi fC_{x}}\right].$$ (19)

Since ϵ≪ *V* subtracting eq (19) from eq (18) one obtains:

$$DF_{m}=DF-DF_{0}=\frac{\alpha }{2\pi fRC_{x}}.$$ (20)

At NIST typical values of *α* are 0.003, 0.0003, −0.0003, −0.003. With a 1 MΩ resistor and a 1000-pF standard capacitor this enables a near full scale test of the dissipation factor on its four ranges. Recent results are shown in table 5. The dissipation factor values are all in units of percent.

Agreement between the calculated values in eq (20) and the corrected measurement *DF*_m_ (the last two columns) are well within ±0.2% of the measured value. This check is performed at approximately 6-month intervals.

It is further proposed that an additional check standard be obtained and measured quarterly. Specifically, a voltage transformer measured regularly at a ratio of 10:1 would give an additional check on the phase angle circuitry and on the bridge windings at something other than a 1:1 ratio.

## 5. Measurement Uncertainties

### 5.1 Voltage Transformers

The records of the National Institute of Standards and Technology show examples of voltage transformers that have been calibrated at 5-year intervals over a period of 30 to 40 years. Invariably the original uncertainty statement covers any variation in ratio correction factor and phase angle observed over this period of time. Voltage transformers are often used by the client in conjunction with other equipment to measure some quantity. For example, used with a current transformer and watthour meter, a voltage transformer can help provide a measure of the energy consumed by a large power transformer. Thus it is important to the clients of this calibration service to obtain a meaningful uncertainty statement that reflects the contribution the voltage transformer would make to their total error budget.

As mentioned earlier in this paper, voltage transformers calibrated at NIST generally fall into two accuracy classes: ±0.03% uncertainty for ratio correction factor, ±0.3 mrad for phase angle; and ±0.01% for ratio correction factor, ±0.1 mrad for phase angle. While it would be possible in some cases to report smaller uncertainties to the clients by more thorough determinations of such parameters as voltage coefficients, proximity effects, and burden dependencies, the present service provides an economical way to present meaningful error statements to the clients and meets their needs.

The analysis of the uncertainties for the ratio correction factor measurements are summarized in table 6. The units are in ppm. The values in parentheses apply to the higher accuracy voltage transformers described in section 2.1. The uncertainties for the phase angle measurement of voltage transformers are the same as is shown in table 6 except the units are microradians instead of ppm.

To calculate the uncertainties reported to the client, the systematic uncertainties tabulated above are algebraically summed and added to three times the root sum of squares of the random uncertainties. The results are shown in table 7.

The values in table 6 are approximate. Some transformers demonstrate stronger voltage dependences than others or stronger burden dependences. In some cases the values in table 7 must be adjusted for such transformers. The purpose of the above tables is to give the users an idea of the sources of errors and how they are used to calculate an uncertainty statement.

Since most of the sources of uncertainty presented in table 6 originate from the transformer under test, NIST could in principle measure a nearly ideal voltage transformer to much better accuracy than shown in table 7. Such a test would be expensive because of the time-consuming care that would be required.

## 5.2 Capacitors

The National Institute of Standards and Technology has the capability to measure the ratio of two capacitors to an estimated systematic uncertainty of ± 1 ppm and ± 1 × 10^−6^ ± 1 % of the measured value for the relative dissipation factor. The values of the standard capacitors used for these comparisons are known to ±10 ppm for capacitance (±1 × 10^−6^ for dissipation factor). The random uncertainty associated with the capacitance measurement is ±1 ppm and ± 1 × 10^−6^ for dissipation factor. Conservatively then, NIST could calibrate a client’s capacitor to an overall uncertainty of ±15 ppm in capacitance and ±5 × 10^−6^ ±1% of the value for dissipation factor. In general, the quoted uncertainty is always larger than this except for low-voltage standard capacitors similar to those used at the National Institute of Standards and Technology. (Low-voltage standard capacitors are in general calibrated elsewhere at NIST. The service described here provides higher voltage calibration of these same capacitors.)

The uncertainty statements for high-voltage standard capacitors and power-factor capacitors depend on the stability of these devices during the course of the NIST measurements. The stability is influenced by both the voltage dependence of the device and self-heating (i.e., the capacitance and dissipation factors vary as the internal energy dissipated heats the device). Self-heating effects are more important for power-factor capacitors. Some power-factor capacitors demonstrate significant hysteresis effects. Assigning an uncertainty statement to these measurements depends on the specific behavior of the capacitor. If self-heating is a problem the calibration report clearly must specify the amount of time the capacitor was energized before the measurement was made. If hysteresis effects are detected they are so noted. Because of the nature of most of these devices, the calibration reports for capacitors usually include a statement of the form: “the estimated uncertainties quoted apply to the above tabulated values and should not be construed as being indicative of the long-term stability of the device under test.” This statement is also important for the compressed gas insulated capacitors whose values might change significantly by handling during shipping.

The actual uncertainty quoted to the client is derived by algebraically summing the systematic uncertainties and adding three times the root mean sum of squares of the random uncertainties. For the capacitance measurement of compressed gas insulated capacitors, the measurement uncertainty will include a 20 ppm contribution because of the possible 1 K variation in temperature of the NIST voltage transformer laboratory. For power-factor capacitors the self-heating variations will dominate ambient temperature effects.

## Figures and Tables

**Table 1:** Measurement capability

Voltage transformers—60 Hz		
Primary voltage 50–170,000 V rms	Secondary voltage >50 V rms	Phase angle <11 mrad
Capacitors—60 Hz		
Applied voltage^a^ 50–170,000 V rms	Capacitance 10pF–0.001 F	Dissipation factor <0.011

a Total power must be less than 50 kVA.

**Table 2:** Gas density dependence

Gas	*∂C/∂P* at *T* = 22.8°C (units of picofarads/pascal)
SF_6_	(2.012±0.022) × 10^−6^+[(5.1±0.6) × 10^−13^]*P*
CO_2_	(0.903±0.015) × 10^−6^+[(1.4±0.4) × 10^−3^]*P*
He	(0.075 ±0.004) × 10^−6^+[(0.2±0.1) × 10^−l3^]*P*

**Table 3:** ANSI standard burdens

ANSI burden	Volt-amperes	Power factor (lagging)
W	12.5	0.10
X	25	0.70
M	35	0.20
Y	7	0.85
Z	200	0.85
ZZ	400	0.85

**Table 4:** Check standard history

Date	Capacitance ratio	Date	Capacitance ratio
6/80	1.000025	10/84	1.000032
6/81	1.000028	4/85	1.000041
9/81	1.000027	6/85	1.000042
1/82	1.000027	10/85	1.000041
4/82	1.000026	12/85	1.000042
7/82	1.000026	1/86	1.000041
9/82	1.000028	5/86	1.000044
1/83	1.000030	7/86	1.000044
3/83	1.000031	10/86	1.000044
6/83	1.000033	2/87	1.000044
8/83	1.000031	7/87	1.000046
12/83	1.000031	12/87	1.000044
1/84	1.000032	4/88	1.000040
5/84	1.000033	11/88	1.000046

**Table 5:** Dissipation factor check standard

Date	α	Measured (*DF*)	Correction (*DF*_0_)	Corrected (*DF*_m_)	Theoretical (*α*/2π*fRC*_x_)
7/82	0.0003	0.08003	0.0002	0.07983	0.07977
	0.003	0.7982	0.0002	0.7980	0.7977
	−0.003	−0.7979	0.0002	−0.7981	−0.7977
	−0.0003	−0.07959	0.0002	−0.07979	−0.07977
3/83	0.0003	0.08135	−0.00014	0.08149	0.08147
	0.003	0.81455	−0.00015	0.8147	0.8147
	−0.003	−0.81475	−0.00015	−0.8146	−0.8147
	−0.0003	−0.08160	−0.00014	−0.08146	−0.08147
10/83	0.0003	0.07952	−0.0001	0.07962	0.07959
	0.003	0.7959	−0.0001	0.7960	0.7959
	−0.003	−0.7960	−0.0001	−0.7959	−0.7959
	−0.0003	−0.07965	−0.0001	−0.07955	−0.07959
1/84	0.0003	0.08174	0.00029	0.08145	0.08143
	0.003	0.8148	0.00029	0.8145	0.8143
	−0.003	−0.8140	0.00029	−0.8143	−0.8143
	−0.0003	−0.08110	0.00029	−0.08139	−0.08143
5/84	0.0003	0.08090	0.0002	0.08070	0.08071
	0.003	0.8076	0.0002	0.8074	0.8071
	−0.003	−0.8071	0.0002	−0.8073	−0.8071
	−0.0003	−0.08050	0.0002	−0.08070	−0.08071
11/84	0.0003	0.08000	0.0000	0.08000	0.07997
	0.003	0.8000	0.0000	0.8000	0.7997
	−0.003	−0.8000	0.0000	−0.8000	−0.7997
	−0.0003	−0.08000	0.0000	−0.08000	−0.07997
4/85	0.0003	0.08060	0.0000	0.08060	0.08059
	0.003	0.8056	0.0000	0.8056	0.8059
	−0.003	−0.8055	0.0000	−0.8055	−0.8059
	−0.0003	−0.08050	0.0000	−0.08050	−0.08059
12/85	0.0003	0.08070	−0.0001	0.08080	0.08071
	0.003	0.8076	−0.0001	0.8077	0.8071
	−0.003	−0.8076	−0.0001	−0.8075	−0.8071
	−0.0003	−0.08070	−0.0001	−0.08060	−0.08071
11/86	0.0003	0.08029	−0.00022	0.08051	0.08046
	0.003	0.8049	−0.00022	0.8051	0.8046
	−0.003	−0.8054	−0.00022	−0.8052	−0.8046
	−0.0003	−0.08067	−0.00022	−0.08045	−0.08046
7/87	0.0003	0.08031	−0.0002	0.08051	0.08045
	0.003	0.8051	−0.0002	0.8053	0.8045
	−0.003	−0.8055	−0.0002	−0.8053	−0.8045
	−0.0003	−0.08071	−0.0002	−0.08051	−0.08045
12/87	0.0003	0.08060	0.0001	0.08050	0.08039
	0.003	0.8053	0.0001	0.8052	0.8039
	−0.003	−0.8051	0.0001	−0.8052	−0.8039
	−0.0003	−0.08040	0.0001	−0.08050	−0.08039
8/88	0.0003	0.08010	−0.0003	0.08040	0.08030
	0.003	0.8037	−0.0003	0.8040	0.8030
	−0.003	−0.8043	−0.0003	−0.8040	−0.8030
	−0.0003	−0.08070	−0.0003	−0.08040	−0.08030
11/88	0.0003	0.08142	0.0000	0.08142	0.08128
	0.003	0.8140	0.0000	0.8140	0.8128
	−0.003	−0.8140	0.0000	−0.8140	−0.8128
	−0.0003	−0.08135	0.0000	−0.08135	−0.08128

**Table 6:** Contributions to uncertainty

	Uncertainties	
	Random	Systematic
Bridge measurement	±2 (±2)	±75 (±25)
Secondary voltage setting		±50 (±10)
Burden setting		±50 (±10)
Transformer self-heating		±75 (±20)
Capacitance ratio measurement	±2 (±2)	± 5 (± 5)

**Table 7:** Total estimated uncertainties

Ratio correction factor	±0.03%	(±0.01%)
Phase Angle	±0.3 mrad	(±0.1 mrad)

## Footnotes

1 Certain commercial products are identified to adequately specify the experimental procedure. In no case does such identification imply recommendation by NIST, nor does it imply the products are the best available.

## 6. Appendix

The voltage transformer will be represented as an ideal transformer with some unknown series output impedance *Z*_0_, as shown in figure 9. The model has been shown to be sufficiently accurate experimentally. The relationship between the input voltage *E*_i_ and the output voltage with zero burden *E*_0_ is:

$$\frac{E_{i}}{E_{0}}=NRCF_{0}e^{-j\Gamma_{0}},$$ (21)

where *N* is the nominal (or turns) ratio of the transformer, *RCF*_0_ is the ratio-correction factor (*N* × *RCF*_0_ = actual ratio) at zero burden, Γ_0_ is the angle by which the secondary voltage vector leads the primary voltage vector, and $j=\sqrt{-1}$. A similar relationship exists between the input voltage *E*_i_ and the output voltage *E*_c_, with secondary burden *C* (having impedance *Z*_c_) shown in figure 10:

$$\frac{E_{i}}{E_{c}}=NRCF_{c}e^{-j\Gamma_{c}}$$ (22)

where *RCF*_C_ is the ratio correction factor with secondary burden *C* and Γ_c_ is the corresponding phase angle.

Equating the current through *Z*_0_ and *Z*_c_ in figure 10, one obtains

$$\frac{E_{0}-E_{c}}{Z_{0}}=\frac{E_{c}}{Z_{c}}$$ (23)

or

$$E_{c}=\frac{E_{0}Z_{c}}{Z_{0}+Z_{c}}.$$ (24)

This can be rewritten in the following form:

$$\frac{E_{i}}{E_{c}}=\frac{E_{i}}{E_{0}}\left(1+\frac{Z_{0}}{Z_{c}}\right).$$ (25)

Setting *Z*_0_ equal to *R*_0_*+jX*_0_ and *Z*_c_ equal to *R*_c_+*jX*_c_, eq (25) becomes:

$$\frac{E_{i}}{E_{c}}=\frac{E_{i}}{E_{0}}\left[1+\frac{R_{0}+jX_{0}}{R_{c}+jX_{c}}\right].$$ (26)

Taking the absolute value of both sides of eq (26), one finds that:

$$\left|\frac{E_{i}}{E_{c}}\right|\approx \left|\frac{E_{i}}{E_{0}}\right|\;\left[1+\frac{R_{0}R_{c}+X_{0}X_{c}}{R_{c}^{2}+X_{c}^{2}}\right],$$ (27)

where it has been assumed that both *R*_0_ and *X*_0_ are much less than *Z*_c_ so that terms of order ${\left[\left(R_{0}R_{c}+X_{0}X_{c}\right)/\left(R_{c}^{2}+X_{c}^{2}\right)\right]}^{2}$ and higher have been neglected. Using eqs (21) and (26), one obtains

$$\frac{E_{i}}{E_{c}}=\left|\frac{E_{i}}{E_{0}}\right|e^{-j\Gamma_{0}}\;\left|1+\frac{R_{0}+jX_{0}}{R_{c}+jX_{c}}\right|$$ (28)

or

$$\frac{E_{i}}{E_{c}}=\left|\frac{E_{i}}{E_{0}}\right|e^{-j\Gamma_{0}}\;\left[1+\frac{\left(R_{0}+jX_{0}\right)\left(R_{c}-jX_{c}\right)}{R_{c}^{2}+X_{c}^{2}}\right].$$ (29)

This can also be expressed as

$$\left|\frac{E_{i}}{E_{c}}\right|e^{-j\Gamma_{c}}=\left|\frac{E_{i}}{E_{0}}\right|e^{-j\Gamma_{0}}\left[\ldots \right].$$ (30)

Both exponentials have arguments much less than one so that discarding quadratic and higher order terms and equating the imaginary components of the left and right sides of eq (10) one obtains

$$\left|\frac{E_{i}}{E_{c}}\right|\Gamma_{c}\approx \left|\frac{E_{i}}{E_{0}}\right|\;\left[\Gamma_{0}+\frac{X_{c}R_{o}-X_{0}R_{c}}{R_{c}^{2}+X_{c}^{2}}\right]$$ (31)

or from eq (5) assuming *Z*_0_≪*Z*_C_

$$\Gamma_{c}\approx \Gamma_{0}-\frac{X_{0}R_{c}-X_{c}R_{0}}{R_{c}^{2}+X_{c}^{2}}.$$ (32)

The resistive and reactive components of the burden *C* can be expressed as

$$R_{c}=\sqrt{R_{c}^{2}+X_{c}^{2}}\cos\theta_{c}$$ (33)

and

$$X_{c}=\sqrt{R_{c}^{2}+X_{c}^{2}}\sin\theta_{c},$$ (34)

where cos*θ*_c_ is the power factor of the burden *C.* From eqs (21) and (22)

$$\left|\frac{E_{i}}{E_{0}}\right|=NRCF_{0}$$ (35)

and

$$\left|\frac{E_{i}}{E_{c}}\right|=NRCF_{c}.$$ (36)

Using eqs (27) and (33)–(36) one obtains

$$RCF_{c}=RCF_{0}\left[1+\frac{1}{\left|Z_{c}\right|}\left(R_{0}\cos\theta_{c}+X_{0}\sin\theta_{c}\right)\right].$$ (37)

For the purposes of this discussion, it will be assumed that burden *C* (having impedance *Z*_c_) above is the burden for which the ratio correction factor and phase angle are to be calculated. The ratio-correction factor and phase angle must be known for some other burden *T*, which shall be designated as having impedance *Z*_t_. Using eq (25) and substituting burden *T* for burden *C:*

$$Z_{0}=\left[\frac{E_{i}/E_{t}}{E_{i}/E_{0}}-1\right]Z_{t}.$$ (38)

or using eq (22)

$$Z_{0}=Z_{t}\left[RCF_{t}e^{-j\left\{\Gamma_{t}-\Gamma_{0}\right\}}-RCF_{0}\right]/RCF_{0}.$$ (39)

Neglecting second order and higher terms

$$Z_{0}\approx Z_{t}\left[RCF_{t}-RCF_{0}+j\left(\Gamma_{0}-\Gamma_{t}\right)\right]/RCF_{0}.$$ (40)

Using the facts that

$$Z_{t}=\left|Z_{t}\right|\left(\cos\theta_{t}+j\sin\theta_{t}\right)$$ (41)

and

$$Z_{0}=R_{0}+jX_{0},$$ (42)

one finds

$$\begin{array}{l} R_{0}\approx \left(\frac{\left|Z_{t}\right|}{RCF_{0}}\right)[\left(RCF_{t}-RCF_{0}\right)\cos\theta_{t}\\ \;+\left(\Gamma_{t}-\Gamma_{0}\right)\sin\theta_{t}] \end{array}$$ (43)

and

$$\begin{array}{l} X_{0}\approx \left(\frac{\left|Z_{t}\right|}{RCF_{0}}\right)\;[\left(\Gamma_{0}-\Gamma_{t}\right)\cos\theta_{t}\\ \;+\left(RCF_{t}-RCF_{0}\right)\sin\theta_{t}]. \end{array}$$ (44)

Using eqs (37), (43), and (44) and the relations:

$$\cos\theta_{c}\;\cos\theta_{t}+\sin\theta_{c}\;\sin\theta_{t}=\cos\left(\theta_{t}-\theta_{c}\right)$$ (45)

$$\cos\theta_{c}\;\sin\theta_{t}-\sin\theta_{c}\cos\theta_{t}=\sin\left(\theta_{t}-\theta_{c}\right)$$ (46)

one finds

$$\begin{array}{l} RCF_{c}\approx RCF_{0}+\left(\frac{B_{c}}{B_{t}RCF_{0}}\right)[\left(RCF_{t}-RCF_{0}\right)\\ \;\cos\left(\theta_{t}-\theta_{c}\right)+\left(\Gamma_{t}-\Gamma_{0}\right)\sin\left(\theta_{t}-\theta_{c}\right)] \end{array}$$ (47)

or

$$\begin{array}{l} RCF_{c}\approx RCF_{0}+\left(\frac{B_{c}}{B_{t}}\right)[\left(RCF_{t}-RCF_{0}\right)\\ \;\cos\left(\theta_{t}-\theta_{c}\right)+\left(\Gamma_{t}-\Gamma_{0}\right)\sin\left(\theta_{t}-\theta_{c}\right)], \end{array}$$ (48)

where *B*_c_ = 1/*Z*_c_ is the burden in Ω^−1^ of the impedance *Z*_c_. Since the second term in eq (47) represents a small correction to the first and since *RCF*_0_ is approximately equal to one, *RCF*_0_ has been dropped from the second term of eq (48). Using eqs (32)–(34)

$$\Gamma_{c}\approx \Gamma_{0}-\frac{1}{\left|Z_{c}\right|}\left(X_{0}\cos\theta_{c}-R_{0}\sin\theta_{c}\right).$$ (49)

Using eqs (43)–(46) and (49)

$$\begin{array}{c} \Gamma_{c}\approx \Gamma_{0}+\left(\frac{B_{c}}{B_{t}RCF_{0}}\right)\;[\left(\Gamma_{t}-\Gamma_{0}\right)\cos\left(\theta_{t}-\theta_{c}\right)\\ \;-\left(RCF_{t}-RCF_{0}\right)\sin\left(\theta_{t}-\theta_{c}\right)] \end{array}$$ (50)

or

$$\begin{array}{c} \Gamma_{c}\approx \Gamma_{0}+\left(\frac{B_{c}}{B_{t}}\right)[\left(\Gamma_{t}-\Gamma_{0}\right)\cos\left(\theta_{t}-\theta_{c}\right)\\ \;-\left(RCF_{t}-RCF_{0}\right)\sin\left(\theta_{t}-\theta_{c}\right)] \end{array}$$ (51)

since *RCF*_0_ is approximately equal to one.

Equations (48) and (51) can be used to calculate the *RCF* and phase angle for some secondary burden *C*, if the ratio correction factors and phase angles are known at some other burden *T*, and at zero burden.
